# Structural Mimics of Recurrent Bell’s Palsy: A Case Report Highlighting Diagnostic Red Flags in Facial Nerve Schwannoma and Metastasis

**DOI:** 10.3390/reports9020178

**Published:** 2026-06-07

**Authors:** Sohyeon Kim, Hyesoo Kwon, Hung Youl Seok

**Affiliations:** 1 Department of Neurology, Dongsan Hospital, Keimyung University School of Medicine, Daegu 42601, Republic of Korea; bjksh@naver.com (S.K.); kwon.hyesoo@gmail.com (H.K.); 2 Department of Neurology, School of Medicine, Kyungpook National University, 130, Dongdeok-ro, Jung-gu, Daegu 41944, Republic of Korea

**Keywords:** facial paralysis, Bell palsy, neurilemmoma, meningeal neoplasms, magnetic resonance imaging, electrophysiology

## Abstract

**Background and Clinical Significance**: Recurrent peripheral facial palsy is most often attributed to idiopathic Bell’s palsy (BP), yet a minority of patients harbor underlying structural lesions. Early recognition of such lesions is essential for timely and appropriate management. **Case Presentation**: We retrospectively reviewed recurrent unilateral facial palsy at a single tertiary center and selected two illustrative patients in whom serial clinical, electrodiagnostic, and magnetic resonance imaging (MRI) findings ultimately indicated structural pathology. The first patient experienced three strictly right-sided episodes over 3.5 years, with worsening House–Brackmann grades (II → III → V) and progressive axonal loss on serial facial nerve conduction studies, culminating in a mass-like enhancing lesion at the geniculate ganglion consistent with schwannoma. The second patient had two left-sided recurrences within seven months. MRI evolved from a subtle intrameatal nodular suspicion without enhancement to a clearly enhancing intrameatal nodule with dural thickening. Additional intracranial nodular enhancements were discovered later and the patient was diagnosed with leptomeningeal metastasis. **Conclusions**: These cases illustrate that strictly ipsilateral recurrences, progressive electrophysiologic deterioration, and atypical or evolving MRI enhancement patterns should prompt thorough re-evaluation for structural causes rather than repeated labeling as idiopathic BP.

## 1. Introduction and Clinical Significance

Bell’s palsy (BP) is the most common cause of acute peripheral facial nerve paralysis, with an annual incidence of 15–30 per 100,000 [1]. The condition is characterized by a sudden onset of unilateral facial weakness resulting from dysfunction of the facial nerve (CN VII), and it accounts for approximately 60–75% of all cases of acute unilateral facial paralysis [1]. Although the pathogenesis remains incompletely understood, reactivation of herpes simplex virus type 1 within the geniculate ganglion is considered the most widely accepted etiological hypothesis [2,3]. The prognosis is generally favorable, with approximately 70–85% of patients achieving complete spontaneous recovery [2]. Recurrent BP (RBP) is less frequent and has been reported in 0.8–19.4% of cases, with a pooled mean recurrence rate of approximately 6.5% based on a systematic review and meta-analysis [4]. Recent studies have identified several risk factors associated with RBP, including diabetes mellitus, hypertension, hypothyroidism, autoimmune disorders, and a family history of BP [5].

However, a subset of patients initially diagnosed with RBP ultimately prove to have structural lesions. In tertiary referral centers, where complex or atypical cases are concentrated, an appreciable proportion of patients initially labeled as recurrent BP are subsequently found to harbor structural lesions such as facial nerve schwannomas, vestibular tumors, or leptomeningeal disease. Recent literature has shown that a proportion of intratemporal facial nerve schwannomas are initially misdiagnosed as BP, highlighting the risk of diagnostic delay [6]. Likewise, in patients with solid malignancies, recurrent facial palsy can be an early manifestation of leptomeningeal metastasis [7]. Despite these concerns, comprehensive descriptions integrating clinical course, serial nerve conduction study (NCS) findings, and MRI changes across multiple episodes remain scarce.

The aim of this report is to describe two illustrative cases of recurrent unilateral facial palsy in which structural lesions—facial nerve schwannoma and leptomeningeal metastasis—were ultimately implicated. By tracking the long-term clinical, electrodiagnostic, and radiologic progress of these patients, we seek to highlight key red flags that should prompt reconsideration of an idiopathic diagnosis and to propose practical implications for the diagnostic approach to recurrent facial palsy. In particular, we aim to address two aspects that have rarely been emphasized in previous literature. First, we introduce the role of the quantitative serial compound muscle action potential (CMAP) amplitude ratio trajectory as a longitudinal diagnostic biomarker, shifting the focus from its traditional use as a single-time-point prognostic measure. Second, we define the concept of a clinico-electrophysiologic discrepancy, where apparent clinical recovery coexists with progressive electrodiagnostic deterioration, as a discrete red flag for structural pathology.

## 2. Case Presentation

This report stems from a retrospective chart review conducted at Keimyung University Dongsan Hospital, in which all patients diagnosed with peripheral facial palsy between September 2018 and February 2026 were screened for recurrent episodes. Recurrent facial palsy was defined as at least two episodes of acute peripheral facial weakness with partial or complete recovery between episodes.

Among these patients, we selected two individuals with recurrent unilateral facial palsy who were ultimately found to have structural causes: one patient with facial nerve schwannoma and the other with leptomeningeal metastasis.

Detailed clinical data, including demographics, comorbidities, episode characteristics, associated symptoms (e.g., pain around the ear, hyperacusis, and altered taste or lacrimation), treatment, and outcomes were reviewed. The severity of facial weakness was graded at each episode using the House–Brackmann (H–B) facial nerve grading system [8]. Serial facial NCS results were reviewed, including lesion pattern (axonal, demyelinating, or mixed) and CMAP amplitude ratios between affected and unaffected sides. MRI findings were analyzed with particular attention to enhancement patterns along facial nerve segments. The episode-by-episode clinical, electrodiagnostic, and radiologic findings for both patients are summarized in Table 1.

### 2.1. Case Descriptions

#### 2.1.1. Case 1

A 48-year-old woman with a history of coronavirus disease 2019 (COVID-19) experienced three episodes of recurrent right-sided facial palsy over approximately 3.5 years. She had no known metabolic or autoimmune comorbidities. The initial episode occurred in June 2022 and was mild, with a H–B grade of II. Facial NCS performed during the episode was normal and MRI revealed enhancement of right facial nerve suspicious of neuritis. She was treated with corticosteroids and antiviral agents, achieving a full recovery to H–B grade I.

The second episode, which occurred 1.6 years later in January 2024, again affected the right side. This time, facial weakness was more pronounced with a H–B grade of III. Facial NCS showed a mixed demyelinating and axonal pattern, with a CMAP amplitude ratio of 2.9:1. Despite the increased clinical severity and electrophysiological changes, the patient again reached good recovery (H–B grade II) following the same treatment.

However, during the third episode in December 2025, facial weakness was severe (H–B grade V) at presentation. Facial NCS demonstrated clear axonal degeneration with a markedly worsened CMAP amplitude ratio of 24:1. MRI obtained during this episode revealed a mass-like enhancing lesion at the right geniculate ganglion, consistent with a facial nerve schwannoma (Figure 1). Following multidisciplinary evaluations, combined facial nerve schwannoma removal and facial reanimation surgery were planned by the department of otolaryngology, given the patient’s persistent severe facial weakness without spontaneous recovery. At present, the patient is awaiting surgery and her facial recovery remains incomplete with a H–B grade of IV.

#### 2.1.2. Case 2

A 55-year-old woman presented with two recurrent episodes of left-sided facial palsy. She had a history of advanced-stage breast cancer, which was terminal at the time of initial diagnosis. She was receiving ongoing systemic chemotherapy at the time of presentation. The initial facial palsy occurred in November 2018. She was managed for BP with a standard regimen of corticosteroids and antiviral agents, achieving full recovery to H–B grade I. A fast imaging employing steady-state acquisition (FIESTA) sequence on MRI revealed a subtle nodular lesion in the left intrameatal segment (Figure 2A). This finding was considered inconclusive, as no clear contrast enhancement was observed (Figure 2B).

Seven months later, in June 2019, she developed a second episode of left-sided facial palsy. She received the same regimen of corticosteroids and antiviral agents as in the first episode, but recovery was partial, reaching H–B grade II. MRI clearly demonstrated a nodular lesion with prominent enhancement in the left intrameatal segment (Figure 2C), accompanied by dural thickening (Figure 2D). While follow-up MRI in 2020 showed partial improvement of the intrameatal lesion, it also revealed new nodular enhancements at other intracranial sites. This multifocal pattern was more consistent with leptomeningeal metastasis. The patient’s systemic condition progressively deteriorated, and she eventually succumbed to breast cancer in May 2020.

## 3. Discussion

These two cases illustrate that recurrent facial palsy may be the initial manifestation of underlying structural lesions, even when clinical presentations initially align with the diagnostic criteria for BP. Therefore, a high index of clinical suspicion is essential for cases showing atypical clinical courses.

Several clinical and paraclinical red flags emerge from these cases. First, strictly ipsilateral recurrences over several years should raise suspicion for structural pathology, especially when the severity of facial weakness tends to worsen with each episode. In Case 1, the presenting H–B grade progressed from II to V over successive attacks. Second, serial NCS is essential to detect subclinical deterioration that may be masked by apparent clinical recovery, as progressive electrodiagnostic decline must prompt repeat contrast-enhanced imaging regardless of favorable clinical recovery. Third, the longitudinal evolution of MRI enhancement patterns proved critical in identifying structural pathology. In idiopathic BP, enhancement is typically linear or segmental and often resolves completely on follow-up imaging [9]. In contrast, our cases exhibited persistence and progression of radiologic findings. In Case 1, the focal mass-like enhancement remained localized and became more distinct over time, serving as a hallmark of facial nerve schwannoma. Notably, Case 2 demonstrated a dynamic transition from a subtle, non-enhancing nodule on initial FIESTA sequences to overt nodular enhancement and subsequent intracranial dissemination. This radiologic progression from localized suspicion to disseminated disease underscores that the persistence or worsening of imaging findings over time is a powerful indicator of structural lesions.

Beyond these established red flags, our report contributes two observations that, to our knowledge, have rarely been emphasized in previous literature on recurrent facial palsy. The most striking is the quantitative trajectory of the CMAP amplitude ratio in Case 1, which evolved from normal to 2.9:1 and ultimately to 24:1 across three episodes. This suggests that the CMAP amplitude ratio may function not only as a prognostic indicator, as it is conventionally regarded, but also as a longitudinal diagnostic biomarker pointing toward underlying structural pathology. Serial quantitative tracking of NCS parameters across multiple recurrent episodes has rarely been documented in the published literature. Equally important is the clinico-electrophysiologic discrepancy itself, which we propose as a discrete conceptual red flag. In Case 1, apparent clinical recovery between the first and second episodes coexisted with continued electrodiagnostic deterioration. This dissociation between subjective clinical improvement and objective electrodiagnostic decline is conceptually distinct from overt progressive clinical severity, and it should prompt reconsideration of an idiopathic diagnosis even when clinical worsening is not yet apparent.

The two cases reported here represent only a small portion of the broader differential diagnosis of recurrent facial palsy. Other structural and non-idiopathic causes that should be considered include benign neoplasms of the facial nerve itself or adjacent structures such as facial nerve schwannoma, vestibular schwannoma, meningioma, and hemangioma; metastatic and leptomeningeal involvement from systemic malignancies; inflammatory and autoimmune disorders such as sarcoidosis, granulomatosis with polyangiitis, and Sjögren syndrome; infectious etiologies including Lyme disease and herpes zoster oticus; and uncommon entities such as Melkersson–Rosenthal syndrome [10]. Our two cases—a facial nerve schwannoma and a leptomeningeal metastasis from breast cancer—are best understood as illustrative examples within this wider spectrum, selected because they highlight specific longitudinal red flags that should prompt reconsideration of an idiopathic diagnosis.

The diagnostic challenges observed in our cases align with existing literature. A recent systematic review highlighted that while 55.6% of intratemporal facial nerve schwannomas present with facial paralysis, approximately 4.5% are initially misdiagnosed as BP [6]. Similarly, malignancy-related leptomeningeal disease is a well-known masquerader that can present with cranial neuropathies resembling idiopathic palsies [7]. Our report extends these findings by integrating detailed serial NCS and MRI data across multiple episodes, illustrating how longitudinal multimodal assessment can eventually unmask the underlying structural etiology. From a practical standpoint, we propose that each recurrent episode of facial palsy should be treated as a distinct diagnostic event rather than being attributed to a simple repetition of idiopathic BP.

Building on this framework, we would like to suggest a few practical situations that should prompt additional work-up in everyday clinical practice. Contrast-enhanced MRI is worth repeating whenever the CMAP amplitude ratio fails to recover, or even worsens, between successive episodes, even when the patient appears to be recovering clinically. A high-resolution temporal bone MRI with thin-slice post-contrast T1 sequences can also be considered once ipsilateral recurrences reach three or more episodes, or when the interval between episodes keeps shortening. When the initial MRI already shows nodular or mass-like enhancement, or enhancement that is confined to a single anatomic segment, repeating the MRI within a few months helps to detect interval growth or evolving enhancement patterns. As a routine adjunct, facial NCS is worth performing at every recurrent episode, so that any cumulative electrodiagnostic deterioration is captured between visits.

This study has several limitations. It describes only two cases from a single center and therefore cannot capture the full spectrum of structural causes of recurrent facial palsy or provide robust estimates of their frequency. Furthermore, the retrospective nature of the study inherently involves incomplete documentation, particularly for early episodes managed at other institutions. The lack of concurrent NCS and MRI for every episode also limited the overall consistency of the longitudinal data. In addition, clinical photographs documenting facial expression at each H–B grade were not available for either patient, as serial photography was not consistently performed during routine outpatient care and retrospective acquisition was not feasible. While facial nerve function at each episode was systematically recorded using the validated H–B grading system, we acknowledge that the absence of photographic documentation limits the visual richness of this report. Nevertheless, the detailed integration of clinical, electrodiagnostic, and radiologic data across episodes offers an instructive view of how structural lesions can mimic RBP.

## 4. Conclusions

Recurrent facial palsy should not automatically be attributed to idiopathic BP, particularly when episodes are strictly ipsilateral, show increasing clinical severity, or are accompanied by clinico-electrophysiological discrepancies. Our cases demonstrate that progressive axonal loss on serial NCS and evolving MRI enhancement are critical red flags for structural etiologies, such as facial nerve schwannoma and leptomeningeal metastasis. Consequently, each recurrence should be managed as a distinct diagnostic event. We recommend a standardized multimodal approach, incorporating serial NCS and follow-up MRI, to ensure the timely identification of underlying structural lesions and to avoid prolonged misclassification as recurrent idiopathic BP.

## Figures and Tables

**Figure 1:**
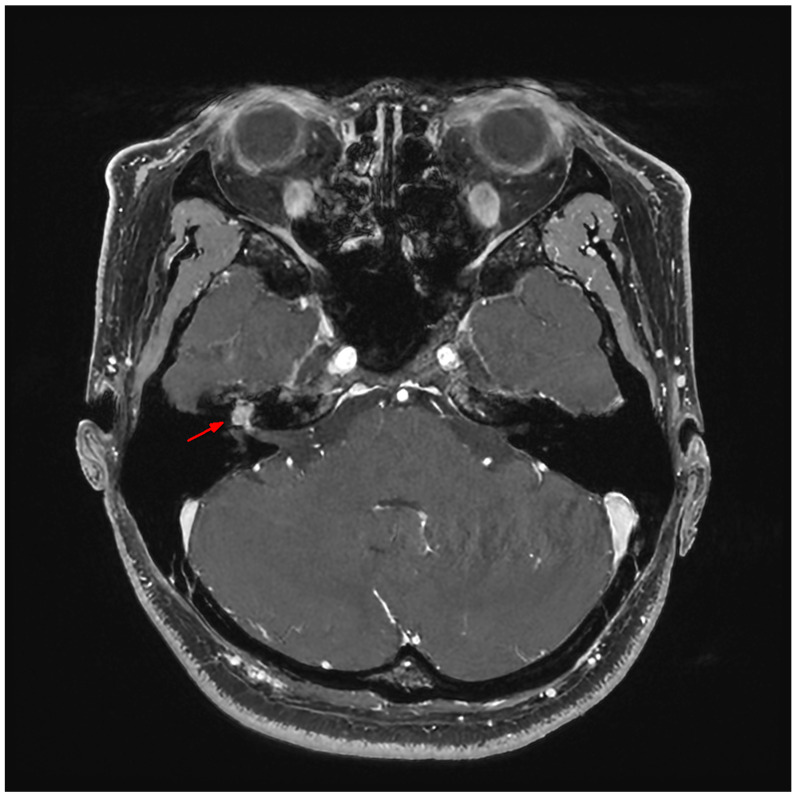
Axial contrast-enhanced T1-weighted image shows a mass-like enhancing lesion at the right geniculate ganglion (arrow), suggestive of a facial nerve schwannoma.

**Figure 2:**
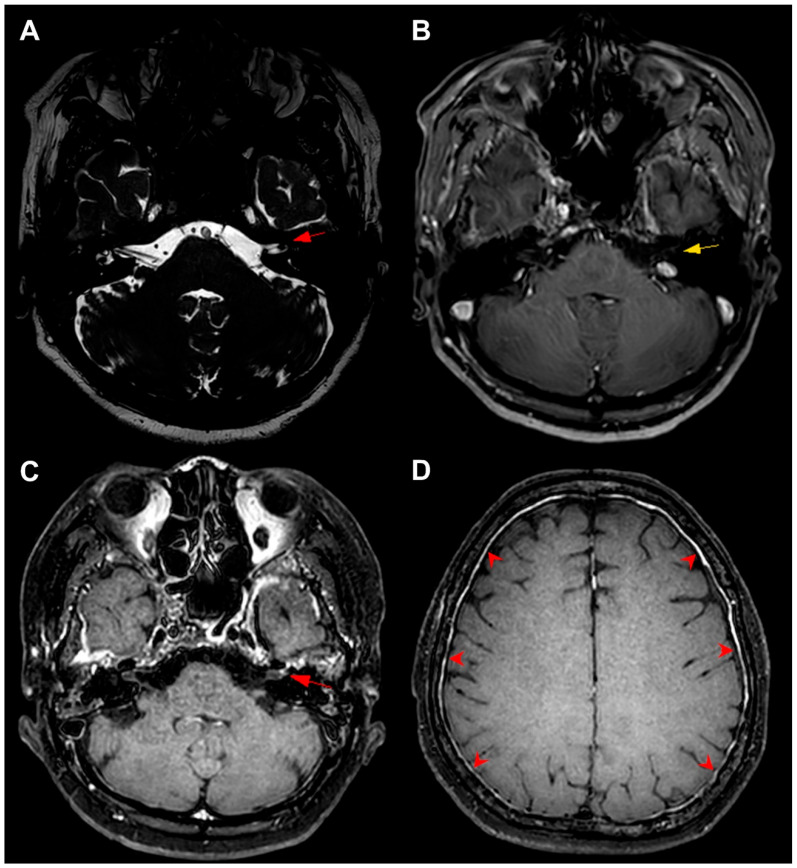
Sequential magnetic resonance imaging (MRI) findings of the patient. (**A**) Axial fast imaging employing steady-state acquisition (FIESTA) sequence during the first episode shows a subtle nodular lesion in the left intrameatal segment (red arrow). (**B**) Corresponding contrast-enhanced T1-weighted image shows no definite enhancement of the nodule (yellow arrow). (**C**) Follow-up contrast-enhanced T1-weighted MRI during the second episode reveals prominent nodular enhancement at the same site (red arrow). (**D**) Contrast-enhanced image at the second episode also demonstrates dural thickening (arrowheads), suggestive of leptomeningeal involvement.

**Table 1:** Episode-by-episode clinical details of patients.

Case No. (Age/Sex)	Ep	Side	Onset	Interval	Initial H–B	Final H–B	Associated Symptoms	MRI Enhancement Segment	MRI Enhancement Morphology	NCS Pattern	CMAP Amplitude Ratio (Unaffected: Affected)
1 (48/F)	1	R	June 2022	-	II	I	None	I + G + Ma	Linear	Normal	1:1
	2	R	January 2024	1.6 yr	III	II	None	N/P	N/P	Axonal + Demyelinating	2.9:1
	3	R	December 2025	1.9 yr	V	IV	None	G	Mass-like	Axonal	24:1
2 (55/F)	1	L	November 2018	-	ND	I	None	I	Nodular (No enhancement)	N/P	N/P
	2	L	June 2019	7 mo	ND	II	None	I	Nodular	Axonal	9:1

CMAP, compound muscle action potential; Ep, episode; F, female; H–B, House–Brackmann grade; L, left; mo, months; ND, not documented; N/P, not performed; R, right; yr, years. Associated symptoms refer to peri-auricular pain, hyperacusis, taste disturbance, and lacrimation disturbance. Enhancement segment abbreviations: I, intrameatal (internal auditory canal); G, geniculate ganglion; Ma, mastoid.

## Data Availability

The data presented in this study are available on request from the corresponding author due to patients’ privacy.
